# Impact of COVID-19 national response on primary care utilisation in Singapore: an interrupted time-series analysis

**DOI:** 10.1038/s41598-024-57142-7

**Published:** 2024-03-17

**Authors:** Vanessa Tan, Gregory Ang, Kelvin Bryan Tan, Cynthia Chen

**Affiliations:** 1 Saw Swee Hock School of Public Health, National University of Singapore and National University Health System, 12 Science Drive 2, #09-01T, Singapore, 117549 Singapore; 2 Department of Statistics and Data Science, National University of Singapore, Singapore, Singapore; 3 Ministry of Health, Singapore, Singapore; 4 Schaeffer Center for Health Policy and Economics, University of Southern California, Los Angeles, USA; 5 Department of Non-Communicable Disease Epidemiology, The London School of Hygiene and Tropical Medicine, London, UK

**Keywords:** Public health, Health policy

## Abstract

Since the start of the pandemic, many national responses, such as nationwide lockdowns, have been implemented to curb the spread of COVID-19. We aim to assess the impact of Singapore’s national responses on primary care utilisation. We performed an interrupted time series using acute and chronic primary care data of 3 168 578 visits between 1 September 2019 and 31 August 2020 over four periods: before any measures were put in place, during Disease Outbreak Response System Condition (DORSCON) Orange, when Circuit Breaker was instituted, and when Circuit Breaker was lifted. We found significant mean reductions in acute and chronic primary care visits immediately following DORSCON Orange and Circuit Breaker. DORSCON Orange was associated with − 2020 mean daily visits (95% CI − 2890 to − 1150). Circuit Breaker was associated with a further − 2510 mean daily visits (95% CI − 3660 to − 1360). Primary care utilisation for acute visits remained below baseline levels even after the Circuit Breaker was lifted. These significant reductions were observed in both acute and chronic visits, with acute visits experiencing a steeper drop during DORSCON Orange. Understanding the impact of COVID-19 measures on primary care utilisation will be useful for future public health planning.

## Introduction

COVID-19 was declared a global pandemic by the World Health Organization (WHO) on 11 March 2020^1^. Although containment measures varied among countries, a wide range of policies, including quarantine orders, border closures and travel restrictions, were enacted to mitigate the spread of the disease. Within healthcare facilities, protocol guidelines such as personal protective equipment and segregation zones were advised to reduce the risk of transmission^2^. However, as the virus continued to evolve and mutate, new waves of infection followed. As of November 2022, over 600 million COVID-19 cases have been reported worldwide^3^.

Singapore was one of the first countries affected by COVID-19, with the index case reported on 23 January 2020^4^. The colour-coded Disease Outbreak Response System Condition (DORSCON) risk assessment was subsequently raised to the second-highest level of Orange on 7 February 2020^5^. Patients with respiratory symptoms were advised to seek medical attention from a primary care practitioner as soon as possible. Early efforts were successful at keeping the infection rate low. However, the emergence of clusters and an increase in unlinked community cases led to a spike in local cases^6^. Singapore instituted a lockdown from 7 April to 31 May 2020 to contain the spread of COVID-19^7^.

Recent studies have quantified the extent of lockdown on the utilisation of healthcare services, including emergency services, hospital admissions and diagnostic procedures^8–10^. For instance, a study in Scotland found that emergency hospital admissions after the nationwide lockdown were 26% lower than in the same period a year before^9^. Another study in a province in China also observed a sharp drop in total hospital visits following the first public health emergency response^10^. Within hospitals, elective surgeries were deferred to reduce the risk of in-hospital transmissions while coping with the surge in positive cases^11^. A global modelling study estimated that over 28 million surgical procedures were deferred during the peak of the pandemic^12^. Measures implemented to contain the disease have caused significant disruption in healthcare services.

Although much attention has been focused on hospital care, the greatest disruption in essential health services was found in primary care and chronic disease management, according to the second WHO National Pulse Survey^13^. As the first point of contact in testing suspected cases, primary care providers had to shift their focus away from the treatment of acute and chronic conditions during the pandemic. Traditionally, primary care services are delivered through in-person consultations. However, face-to-face consultations had to be postponed, particularly for those with underlying medical conditions as they were at higher risk of developing serious complications^14,15^. In addition, other primary care services such as preventive care screenings had to be suspended to prioritise the safety of patients and healthcare workers^16^. To date, limited studies have evaluated the impact of COVID-19 interventions on face-to-face delivery of primary care services.

Primary care in Singapore is offered by a network of polyclinics, which are public primary care clinics that provide subsidised care, and private general practitioner clinics. In this study, we examine the impact of the COVID-19 national response on primary care utilisation by measuring the impact of (1) DORSON Orange and (2) Circuit Breaker on acute and chronic public primary care visits. Understanding the impact of COVID-19 measures can help primary care to better prepare for future public health emergencies.

## Methods

### Study population and periods

Daily aggregated visit data from all polyclinics were extracted from the Singapore Ministry of Health administrative database from September 2019 to August 2020. Visits were categorised as acute or chronic visits. As polyclinics are open on weekdays, with half working days on Saturdays and the eve of public holidays, we excluded visits on Saturdays and the eve of public holidays.to minimise large fluctuations in daily visits.

#### DORSCON Orange (7 February 2020–25 April 2022)

Singapore uses a colour-coded DORSCON framework during a disease outbreak. The DORSCON framework was first introduced during the H1N1 outbreak in 2008^17^. It comprises four progressive degrees of national response depending on the severity and spread of the infectious disease. On 7 February 2020, two weeks after the index case, the risk assessment was raised to the second highest level of Orange, suggesting that the outbreak has a moderate to high public health implication^5^.

Non-essential large-scale events with more than 1000 attendees were cancelled or deferred. Precautionary measures such as mandatory daily temperature monitoring and safe distancing were implemented in workplaces and schools. All short-term visitors were banned from entering or transiting through Singapore to reduce the risk of imported cases. As the number of cases started to rise, social distancing measures were stepped up.

During this period, primary care providers were the first point of contact in screening for suspected COVID-19 patients before they were referred to secondary and tertiary hospitals for further management. Segregation zones were set up to reduce the risk of transmission from high-risk patients^18^. Measures were put in place to mitigate the risk of cross-infection between patients by limiting the number of visitors to prevent overcrowding and by active health surveillance of frontline staff^18^. In addition, healthcare workers were required to don full personal protective equipment when caring for suspected or confirmed cases^18^.

#### Circuit Breaker (7 April 2020–31 May 2020)

On 7 April 2020, the government instituted a Circuit Breaker lockdown to curb the rising transmission rate^7^. Except for essential services such as healthcare, social services, food, transportation and financial services that supported daily needs, all other workplaces were closed^19^. Schools shifted to full home-based learning with co-curricular activities suspended^19^. All social gatherings were also prohibited. Residents were advised to stay home and only head out for essential services. On 21 April 2020, measures were further tightened where the list of essential businesses allowed to operate was further reduced and mask-wearing became mandatory^20^.

Primary care services in some public clinics, which include all five polyclinics in this study, were reorganised into essential services which include general medical consultation and non-essential services such as physiotherapy and minor elective procedures^21^. Non-essential services were deferred while essential services were scaled down where possible^21^. To limit potential exposure to suspected cases, medication delivery services were offered to patients with stable chronic conditions^21^. This reduced the patient load and helped to conserve manpower to be redeployed to other COVID-19 facilities.

The lockdown was lifted on 1 June 2020. Towards the end of the Circuit Breaker, activities were allowed to resume gradually in phases as the community transmission rate remained under control^22^. The first phase involved a gradual and cautious reopening of the economy. Essential services continued and selected businesses and activities were allowed to resume with strict safety measures in place. The second phase further relaxed restrictions, allowing a wider range of activities and businesses to resume operations. However, precautionary measures such as safe distancing and mask-wearing remained in force. The third phase represented a new normal with more activities resuming and larger gatherings permitted. Strict health protocols continued, but there was a greater degree of normalcy compared to the earlier phases.

### Statistical analysis

Descriptive statistics were used to summarise patient visit data. We employed an interrupted time series analysis, accounting for autocorrelation among daily visits for all polyclinics by fitting segmented linear regression models, estimated using generalised least squares estimation with autocorrelated errors^23^. We commenced the data series in September 2019, five months before the risk assessment was raised to DORSCON Orange. The segments modelled were 1 September 2019 to 6 February 2020 (Baseline), 7 February 2020 to 6 April 2020 (DORSCON Orange), 7 April 2020 to 31 May 2020 (Circuit Breaker), and 1 June 2020 to 31 August 2020 (Post Circuit Breaker). Briefly, we compared total primary care visits with the previous period to estimate a level and trend change.

Demographic information (daily average age of all patients in years by visit type) was extracted from the electronic medical records. As there may be differences in primary care utilisation across age groups by visit type, we adjusted the model with daily mean patient age by visit type. Day of the week dummy variables were included in the model to account for variability in daily clinic visits between weekdays. We included three nested models in our analysis. The first model was the unadjusted model. The second model adjusted for the days of the week, and the third model adjusted for days of the week and daily mean patient age by visit type. Clinic visits were also stratified based on visit type (acute or chronic), and separate ITS models were fitted to each visit type. Further details on the model specification and autocorrelation were included in the appendix (Supplementary Appendix). All analyses were conducted in R version 3.6.1^24^. The statistical significance threshold was set at 0.05.

## Results

Table 1 presents the patient characteristics for visits between 01 September 2019 and 31 August 2020. There were 3,168,578 chronic or acute clinic visits during this period from all polyclinics in Singapore. Across the different periods, patients who sought primary care were generally older during Circuit Breaker (Baseline: 48.5 ± 9.16 years; DORSCON Orange: 48.9 ± 10.1 years; Circuit Breaker: 52.3 ± 8.55 years; Post Circuit Breaker: 50.5 ± 9.07 years). The proportion of patients who sought chronic care was higher during Circuit Breaker than in other periods (Baseline: 57.4%; DORSCON Orange: 60.8%; Circuit Breaker: 71.2%; Post Circuit Breaker: 68.4%).

**Table 1:** Patients visit characteristics during Baseline, DORSCON Orange, Circuit Breaker, and Post Circuit Breaker.

	Overall	Baseline	DORSCON Orange	Circuit Breaker	Post Circuit Breaker
	01 Sep ‘19–31 Aug ‘20	01 Sep ‘19–06 Feb ‘20	07 Feb ’20–06 Apr ‘20	07 Apr ’20–31 May ‘20	01 Jun ’20–31 Aug ‘20
Number of visits, n	3 168 578	1 641 645	497 047	306 562	723 324
Average daily age	49.6 ± 9.29	48.5 ± 9.16	48.9 ± 10.1	52.3 ± 8.55	50.5 ± 9.07
Visit type, n (%)					
Acute	1 211 196 (38.2%)	699 735 (42.6%)	194 967 (39.2%)	88 224 (28.8%)	228 270 (31.6%)
Chronic	1 957 382 (61.8%)	941 910 (57.4%)	302 080 (60.8%)	218 338 (71.2%)	495 054 (68.4%)

Data are mean ± SD or n (%) unless otherwise stated. The Baseline period was defined from 1 September 2019 to 6 February 2020; DORSCON Orange was defined from 7 February 2020 to 6 April 2020; Circuit Breaker period was defined from 7 April 2020 to 31 May 2020; Post Circuit Breaker period was defined from 1 June 2020 to 31 August 2020.

**Figure 1:**
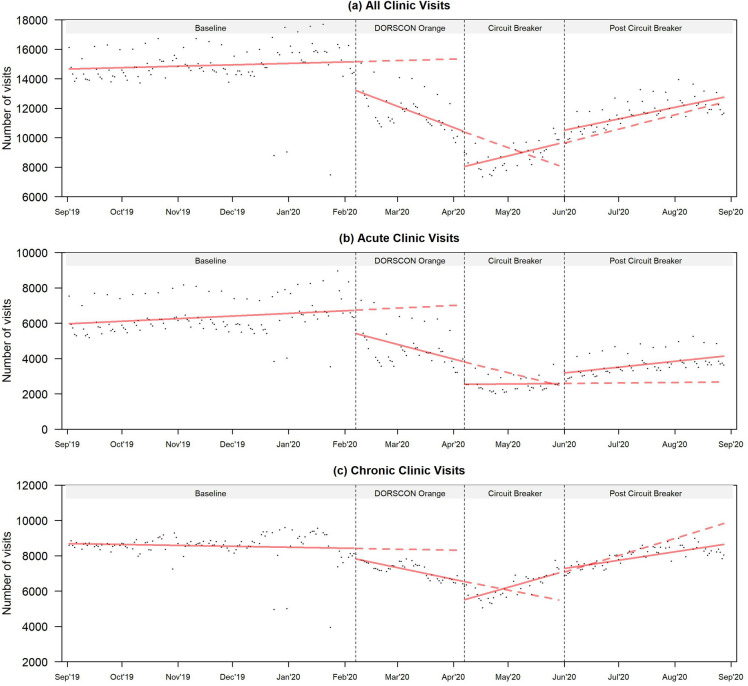
Fitted lines of unadjusted segmented regression models for (**a**) both acute and chronic, (**b**) acute and (**c**) chronic clinic visits. Points represent the observed daily clinic visits from September 2019 to August 2020. The dotted vertical lines represent the three interruption time points: DORSCON Orange (7 February 2020), Circuit Breaker (7 April 2020), and Post Circuit Breaker (1 June 2020). The solid fitted lines represent the unadjusted segmented regression model of clinic visits at each period. The dotted fitted lines represent the expected clinic visits based on the preceding period, had the interruption not occurred. Singapore remained in DORSCON Orange until 25 April 2022.

Unadjusted and adjusted estimates of the daily total, acute and chronic visits are shown in Table 2. Estimates from the unadjusted models are also shown in Fig. 1. Visual inspection of the autocorrelation and partial autocorrelation plots indicated the presence of autocorrelation for all the models (Supplementary Figs. S1–S3). Overall, there was a decrease in daily clinic visits throughout the study period, with acute visits experiencing a steeper drop compared to chronic visits (Fig. 1).

**Table 2:** Interrupted time-series analysis of daily total, acute and chronic polyclinic visits.

	Baseline		DORSCON Orange			Circuit Breaker			Post Circuit Breaker		
	Intercept	Trend	Level change, against Baseline	Trend	Difference in trends (DORSCON Orange—Baseline)	Level change, against DORSCON Orange	Trend	Difference in trends (Circuit Breaker—DORSCON Orange)	Level change, against Circuit Breaker	Trend	Difference in trends (Post Circuit Breaker –Circuit Breaker)
Unadjusted											
Total	14,700*** [14,200, 15,200]	4 [− 3, 12]	− 1960*** [− 2880, − 1050]	− 64*** [− 92, − 35]	− 71*** [− 104, − 39]	− 2330*** [− 3460, − 1200]	46* [9, 83]	113*** [62, 163]	864 [− 201, 1930]	36*** [20, 52]	− 8 [− 52, 35]
Acute	5970*** [5650, 6280]	6** [2, 11]	− 1320*** [− 1890, − 759]	− 38*** [− 57, − 19]	− 45*** [− 65, − 25]	− 1270*** [− 1960, − 569]	1 [− 23, 25]	39* [8, 70]	599 [− 59, 1 260]	15** [4, 26]	14 [− 12, 41]
Chronic	8690*** [8410, 8970]	− 2 [− 6, 2]	− 627* [− 1150, − 101]	− 30** [− 50, − 10]	− 27** [− 46, − 8]	− 1080** [− 1740, − 415]	45*** [19, 71]	75*** [45, 106]	240 [− 382, 863]	22*** [10, 33]	− 23 [− 50, 2]
Adjusted^a^											
Total	13, 500*** [6940, 20,000]	4 [− 2, 12]	− 2 020*** [− 2890, − 1150]	− 71*** [− 104, − 38]	− 72*** [− 104, − 40]	− 2 510*** [− 3660, − 1360]	54* [12, 95]	123*** [73, 172]	565 [− 464, 1 590]	41*** [21, 60]	− 14 [− 57, 28]
Acute	9040*** [5950, 12,100]	7** [2, 11]	− 1480*** [− 2050, − 907]	− 36*** [− 55, − 17]	− 43*** [− 63, − 23]	− 1040** [− 1780, − 304]	14 [− 10, 38]	50** [19, 81]	114 [− 562, 790]	15** [4, 27]	1 [− 25, 28]
Chronic	− 21,100*** [− 27,100, − 15,000]	− 4* [− 8, 0]	− 1010*** [− 1460, − 557]	− 33*** [− 48, − 18]	− 28*** [− 44, − 13]	− 1460*** [− 2020, − 902]	52*** [32, 71]	85*** [60, 110]	258 [− 263, 779]	33*** [24, 42]	− 19 [− 40, 2]

^a^Models adjusted for days of the week and daily patient average age. Both models accounted for autocorrelation.

Data are β-coefficients [95% confidence intervals] from the generalised least squares linear regression model.

*p < 0.05, ** *p* < 0.01, *** *p* < 0.001.

### All visits

The adjusted model showed increasing visits for all visits prior to DORSCON Orange (Table 2). DORSCON Orange was associated with an immediate reduction of − 2020 visits (95% Confidence Interval [CI] − 2890 to − 1150). Following DORSCON Orange, there was a decreasing trend in all clinic visits of − 71 visits per day (95% CI − 104 to − 38). This was in contrast with the increasing trend before DORSCON Orange (difference in trend between DORSCON Orange and Baseline: − 72 visits per day; 95% CI − 104 to − 40).

Circuit Breaker was associated with an immediate reduction of –2510 visits (95% CI − 3660 to − 1360). Following Circuit Breaker, daily clinic visits increased at a rate of 54 visits per day (95% CI 12–95). This was significantly different from the trend during DORSCON Orange (difference in trend between Circuit Breaker and DORSCON Orange: 123 visits per day; 95% CI 73–172).

The lifting of Circuit Breaker was associated with a non-significant increase of 565 visits (95% CI − 464 to 1 590). The increasing trend in clinic visits during Post Circuit Breaker was not as steep (41 visits per day; 95% CI 21–60), but was not significantly different compared to Circuit Breaker (difference in trend between Post Circuit Breaker and Circuit Breaker: − 14 visits per day; 95% CI − 57 to 28).

### By visit types

Before DORSCON Orange, there was a modest increase in mean daily acute and chronic visits respectively (Table 2). DORSCON Orange was associated with an immediate reduction of − 1480 acute visits (95% CI − 2050 to − 907) and − 1010 chronic visits (95% CI − 1460 to − 557). Following DORSCON Orange, there was a steeper decrease in acute visits compared to chronic visits (− 36 acute visits per day (95% CI − 55 to − 17); − 33 chronic visits per day (95% CI − 448 to − 18)), which were both significantly different from the trend in Baseline.

There was also a reduction in acute and chronic visits following Circuit Breaker, with chronic visits experiencing a larger reduction in the adjusted model. Circuit Breaker was associated with a significant reduction of –1 040 acute visits (95% CI − 1780 to − 304) and − 1460 chronic visits (95% CI − 2020 to − 902). In the unadjusted model, a greater reduction in acute visits was observed (acute visits: − 1 270 visits, 95% CI − 1960 to − 569; chronic visits: − 1080 visits, 95% CI − 1740 to − 415). Across visit types, the trend during Circuit Breaker also differed, with chronic visits experiencing a steeper increase in daily visits compared to acute visits (14 acute visits per day (95% CI − 10 to 38); 52 chronic visits per day (95% CI 32–71)).

The easing of Circuit Breaker was associated with a non-significant increase in acute visits of − 114 daily visits (95% CI − 562 to 790) and a non-significant increase in chronic visits of 258 daily visits (95% CI − 263 to 779). This was followed by a significant increasing trend in acute daily visits of 15 per day (95% CI 4–27). The trend in acute visits during Post Circuit Breaker was not significantly different from the trend during Circuit Breaker (difference in mean acute visits trend between Circuit Breaker and Post Circuit Breaker: 1, 95% CI − 25 to − 28). The trend in chronic visits during Post Circuit Breaker was also not significantly different from the trend during Circuit Breaker (difference in mean chronic visits trend between Circuit Breaker and Post Circuit Breaker: − 19, 95% CI − 40 to 2). Compared to acute visits, chronic visits experienced a steeper increase in daily visits during Post Circuit Breaker (33 chronic visits per day; 95% CI 24–42).

## Discussion

Following early implementation and national responses to suppress the spread of COVID-19, Singapore reported one of the lowest mortality rates in the world^25^. Having experienced two pandemics previously, the severe acute respiratory syndrome (SARS) in 2003 and influenza A (H1N1) in 2008, the government developed the DORSCON risk assessment to facilitate containment measures across sectors^26^. The government responded swiftly by activating the risk assessment to the second highest level of DORSCON Orange just 15 days after the first case was reported. Early efforts to contain the virus focused on reducing the risk of transmission.

At the start of the pandemic, the public was advised to exercise social responsibility if feeling unwell by seeking medical attention immediately. To ensure primary care remains accessible and affordable in times of national emergency, the government activated the Public Health Preparedness Clinics (PHPC) scheme involving more than 900 general practitioners on 18 February 2020^27^. In addition to polyclinics, patients with respiratory symptoms were offered subsidised treatment and medications at PHPC, where the wait times are usually shorter. This reduces the load of patients with acute conditions on polyclinics as similar treatment options were available at PHPC. At the same time, pre-emptive measures were also put in place. Patients with respiratory symptoms were issued with mandatory five days of sick leave and they were legally required to stay home and only leave to seek additional medical attention^28^. However, for patients who were sick but had work attendance incentives tied to sick leave, this policy could have deterred them from seeking treatment, overall reducing the number of acute visits^29^.

Non-pharmaceutical interventions, such as mask-wearing, good hygiene practices and social distancing, were also encouraged to reduce the transmission of COVID-19. These measures were found to reduce the transmission of other viral respiratory infections with similar modes of transmission as COVID-19^30,31^. Additionally, travel restrictions also limited the spread of other respiratory infections across national borders^32^. In Singapore, the implementation of non-pharmaceutical interventions was associated with a reduction in the prevalence of respiratory viruses such as influenza, which consistently remained low until the end of 2020^33^.

In the early stages of the pandemic, primary care was used to test for suspected cases before they were referred to hospitals for further treatment. Despite efforts to mitigate the risk of cross-infection between patients by setting up segregation zones and triaging patients by their COVID-19 risk profile, patients may be reluctant to visit the doctor lest they be exposed to infected cases^34^. A study conducted in Singapore revealed that 40% of patients with chronic conditions missed their healthcare appointments during the outbreak, with 72% doing so voluntarily due to a greater perceived risk of infections at a healthcare institution^35^. This sentiment was also prevalent in other countries^36^. Studies elsewhere have shown that patients with underlying chronic conditions did not seek medical care for fear of exposure to COVID-19^37^. These could have led to a drop in overall primary care visits.

As the number of cases started to spike, the government imposed Circuit Breaker to keep cases under control. The public was advised to avoid going out unless necessary as work-from-home arrangements became the default and schools shifted to home-based learning. Non-essential services were deferred while essential services were scaled down whenever possible. For patients who required medication refills, these were done through a medication delivery service if applicable^38^. All social gathering events were also banned, which reduced the spread of acute respiratory infections.

Our analysis revealed a contrasting pattern in the reduction of acute and chronic visits associated with Circuit Breaker in the unadjusted and adjusted models. In the unadjusted model, we observed a larger reduction in acute visits, while the adjusted model showed a greater reduction in chronic visits. Notably, patients were 0.85 years older during Circuit Breaker compared to DORSCON Orange (average age: 60.9 vs. 60.1 years, *p* < 0.001), a demographic factor that likely contributed to the increased reduction in chronic visits in the adjusted model. This divergence in the reduction of acute and chronic visits, evident across both models, highlights the vulnerability of specific patient populations, particularly those older and with chronic conditions. This underscores the need for targeted interventions and strategic resource allocation during public health crises.

During this period, there was also a push for telehealth services^39^. This may have resulted in the conversion of some face-to-face primary care visits from polyclinics to telehealth visits, which could have freed up some of the appointments in polyclinics to be reallocated to patients with chronic conditions. Towards the end of Circuit Breaker, primary healthcare services in hospitals were allowed to resume in phases where patients with chronic medical conditions were attended to first to ensure continuity of chronic care^22^. This might also have encouraged patients with chronic medical conditions to seek care in polyclinics, as the fear of seeking primary care subsided. Thus, the proportion of daily chronic visits appears to increase faster than acute visits during Circuit Breaker.

Similar findings have been observed in other countries. Following the lockdown in the UK, there was a significant reduction in virtual and face-to-face primary care consultations related to specific health conditions, including acute respiratory and cardiovascular conditions^40^. Three months after the restriction was lifted, remote and in-person consultations were still lower than pre-lockdown levels. Other studies conducted in the UK also reported substantial reduction with slow recovery in primary care attendance associated with asthma exacerbation and chronic obstructive pulmonary disease^41–43^. The authors hypothesise that the reduction in primary care visits may have been due to the reprioritisation of primary health services in which general practitioners (GPs) were required to balance COVID-19 infection care with primary care services coupled with fears associated with COVID-19 infection. To protect the patients, GPs were advised to minimise the number of in-person consultations. Across the world, healthcare services for other conditions were scaled back as resources were redirected to care for COVID-19 cases. This has caused delays in healthcare delivery for other conditions. This delay or avoidance of seeking care can increase morbidity and mortality^44^.

There are limitations to this study. The data used in this study is limited to a cluster of public primary care clinics. Primary healthcare services in Singapore are delivered through a network of public primary care clinics and private general practitioner clinics. At the time of this study, 20 public primary care clinics were in operation, comprising only 20% of the sector^45^. Additionally, the distribution of chronic care needs addressed by public clinics is significantly imbalanced, with 80% of chronic care needs addressed by public care clinics^45^. Likewise, the proportion of acute care needs addressed by private clinics is much higher. Furthermore, telemedicine played a crucial role in providing primary care services during the pandemic while minimizing physical contact. The inherent variation in attendance patterns between public clinics, private clinics, and telemedicine may introduce complexities in generalizing the findings across the primary care landscape in Singapore.

While our study shed light on the impact of DORSCON Orange and Circuit Breaker on primary care utilisation, the impact may not be directly attributable to these policies as there were other nationwide measures concurrently rolled out such as public education and enforcement of non-pharmaceutical interventions. Additionally, the reprioritisation of primary care services also affected other primary care services that were not examined in this study. Moreover, as the relaxation of the Circuit Breaker measures occurred gradually in a phased approach, our model may only partially encapsulate the complete impact of these policies on primary care visits.

Lastly, primary care manages more than just acute and chronic medical conditions; it includes preventive health screening, immunisation, and dental services.

Despite these limitations, this study provides an understanding of primary care utilisation in the face of the COVID-19 national response. The unintended effect of restrictive measures may have been overlooked and understanding it can help inform future policy discussions on balancing infectious disease care and essential primary care services.

Our findings add to the growing body of literature on the impact of the COVID-19 national response on healthcare utilisation. Understanding the impact of national responses on primary care is especially crucial as primary care serves as the first point of contact with patients, not just in the face of COVID-19 but also in the growing burden of chronic conditions. It is important to recognise the challenges that other patients may face. Disruption in essential primary care services, particularly chronic care management, may lead to profound health consequences. Further studies with a longer observation period may be needed to understand the prolonged impact of COVID-19.

### Ethical approval

The study was approved by the ethics committee of the National University of Singapore Institutional Review Board (NUS-IRB-2021-611). All methods were carried out in accordance with relevant guidelines and regulations. Informed consent was obtained from all subjects and/or their legal guardian(s).

### Supplementary Information

Supplementary Information.

## Data Availability

The data that support the findings of this study are available from the Ministry of Health but restrictions apply to the availability of these data, which were used under license for the current study, and so are not publicly available. Data are however available from the authors upon reasonable request and with permission of the Ministry of Health through Iris Tham from the TRUST Data Concierge, (trust_data_concierge@moh.gov.sg).

## Abbreviations

COVID-19: Coronavirus disease of 2019
DORSCON: Disease Outbreak Response System Condition
GPs: General practitioners
H1N1: Influenza A
PHPC: Public Health Preparedness Clinics
SARS: Severe acute respiratory syndrome
WHO: World Health Organization
