# Supplementary material for: Impact of COVID-19 national response on primary care utilisation in Singapore: an interrupted time-series analysis
Source: Sci Rep. 2024 Mar 17;14:6408. doi: 10.1038/s41598-024-57142-7 (PMC10944837; doi:10.1038/s41598-024-57142-7)

Supplementary Appendix

(I) Model Specification

This study evaluated three interruptions with four segments, each with its own slope.

$$Y_t = \beta_0 + \beta_1 T_t + \beta_2 W_t + \beta_3 W_t(T_t - t_{Baseline}) + \beta_4 X_t + \beta_5 X_t(T_t - t_{preCB}) + \beta_6 Z_t + \beta_7 Z_t(T_t - t_{CB}) + \epsilon_t$$

where,
Y_t is the aggregated daily outcome (polyclinic visits);
T is the time since the start of the study (coded 0, 1, 2, …, t);
$W = \{0,1\}$, 0 for the days between 1st September 2019 to 6th February 2020 and 1 for the days between 7th February 2020 to 31st August 2020;
$X = \{0,1\}$, 0 for the days between 1st September 2019 to 4th April 2020 and 1 for the days between 5th April 2020 to 31st August 2020;
$Z = \{0,1\}$, 0 for the days between 1st September 2019 to 31st May 2020 and 1 for the days between 1st June 2020 to 31st August 2020.
$t_{Baseline} = 107$, the time on the last day of the Baseline period since the start of the study.
$t_{preCB} = 149$, the time on the last day of the Pre Circuit Breaker period since the start of the study.
$t_{CB} = 181$, the time on the last day of the Circuit Breaker period since the start of the study.

The parameters that are estimated are:
β_0, the intercept which represents the mean daily polyclinic visits on 1st September 2019.
β_1, the slope (change in mean daily polyclinic visits) during baseline.
β_2, the step change in mean daily polyclinic visits immediately following DORSCON Orange.
β_3, the difference in slope between Baseline and Pre Circuit Breaker. The slope of Pre Circuit Breaker is calculated as $\beta_1 + \beta_3$.
β_4, the step change in mean daily polyclinic visits immediately following Circuit Breaker.
β_5, the difference in slope between Pre Circuit Breaker and Circuit Breaker. The slope of Circuit Breaker is calculated as $\beta_1 + \beta_3 + \beta_5$.
β_6, the step change in mean daily polyclinic visits immediately after Circuit Breaker was lifted.
β_7, the difference in slope between Circuit Breaker and Post Circuit Breaker. The slope of Post Circuit Breaker is calculated as $\beta_1 + \beta_3 + \beta_5 + \beta_7$.

We additionally adjusted for several covariates in our models. The full specification of these models is as follows:

$$Y_t = \beta_0 + \beta_1 T_t + \beta_2 W_t + \beta_3 W_t(T_t - t_{preCB}) + \beta_4 X_t + \beta_5 X_t(T_t - t_{CB}) + \beta_6 Z_t + \beta_7 Z_t(T_t - t_{postCB}) + \beta_8\, Tuesday + \beta_9\, Wednesday + \beta_{10}\, Thursday + \beta_{11} Friday + \beta_{12} Average\ Age + \epsilon_t$$

where,
Tuesday was 1 if the date falls on a Tuesday and 0 otherwise;
Wednesday was 1 if the date falls on a Wednesday and 0 otherwise;
Thursday was 1 if the date falls on a Thursday and 0 otherwise;
Friday was 1 if the date falls on a Friday and 0 otherwise;
Average Age was entered as a continuous variable.

See the main document for an explanation of why these covariates were included.

(II) Selection of Lags

Autocorrelation was detected by visually inspecting the autocorrelation correlation function (ACF) and partial autocorrelation function (PACF) plots (Supplementary Figures S1, S2, S3). For models with significant autocorrelation, different autocorrelation parameters were tested. The final selection of autocorrelated terms was chosen by selecting the model with the lowest Akaike Information Criterion (AIC).

Supplementary Figure S1. Autocorrelation Function (ACF) and Partial Autocorrelation Function (PACF) plots for acute and chronic clinic visits.

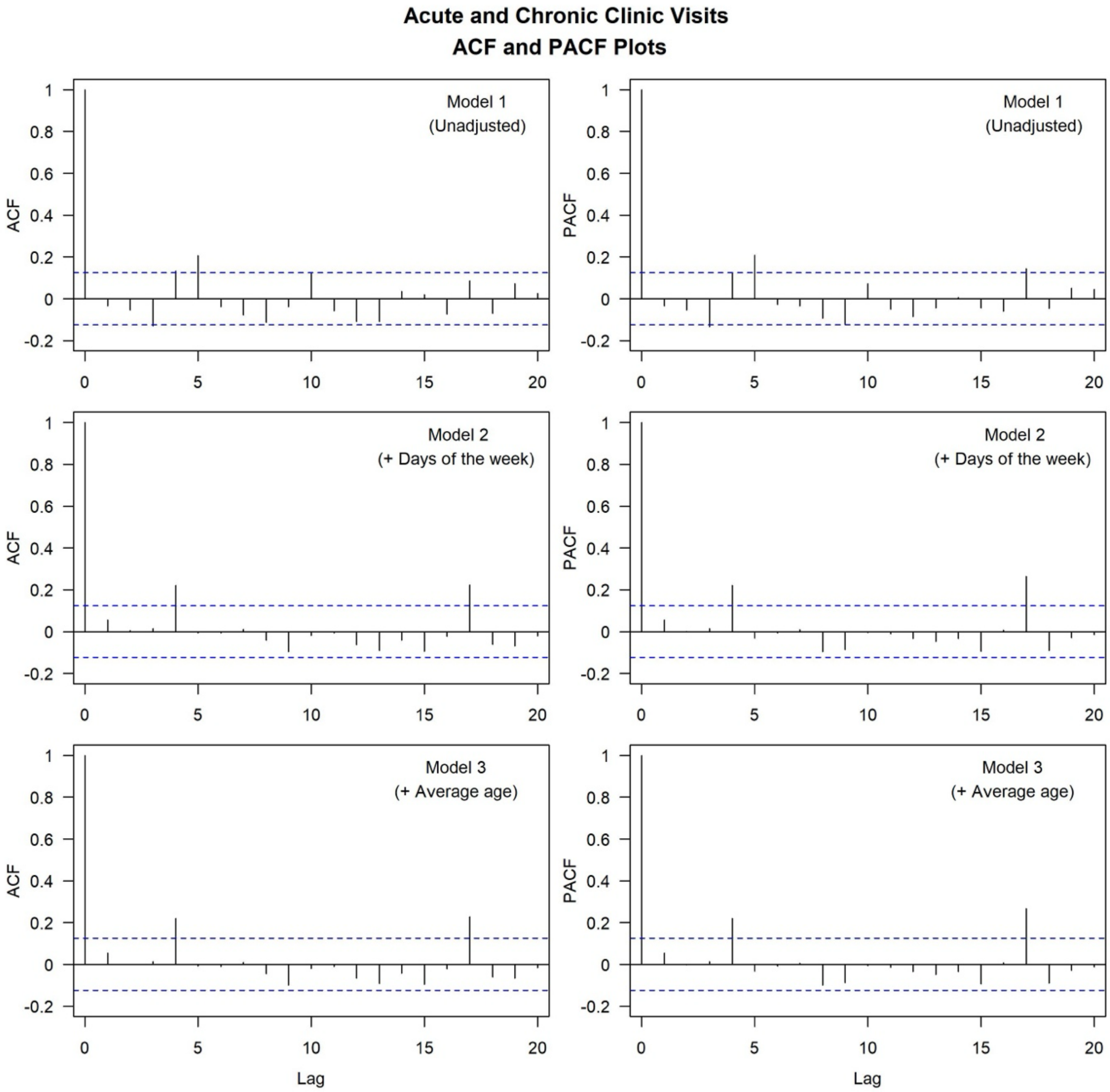

Supplementary Figure S2. Autocorrelation Function (ACF) and Partial Autocorrelation Function (PACF) plots for acute clinic visits.

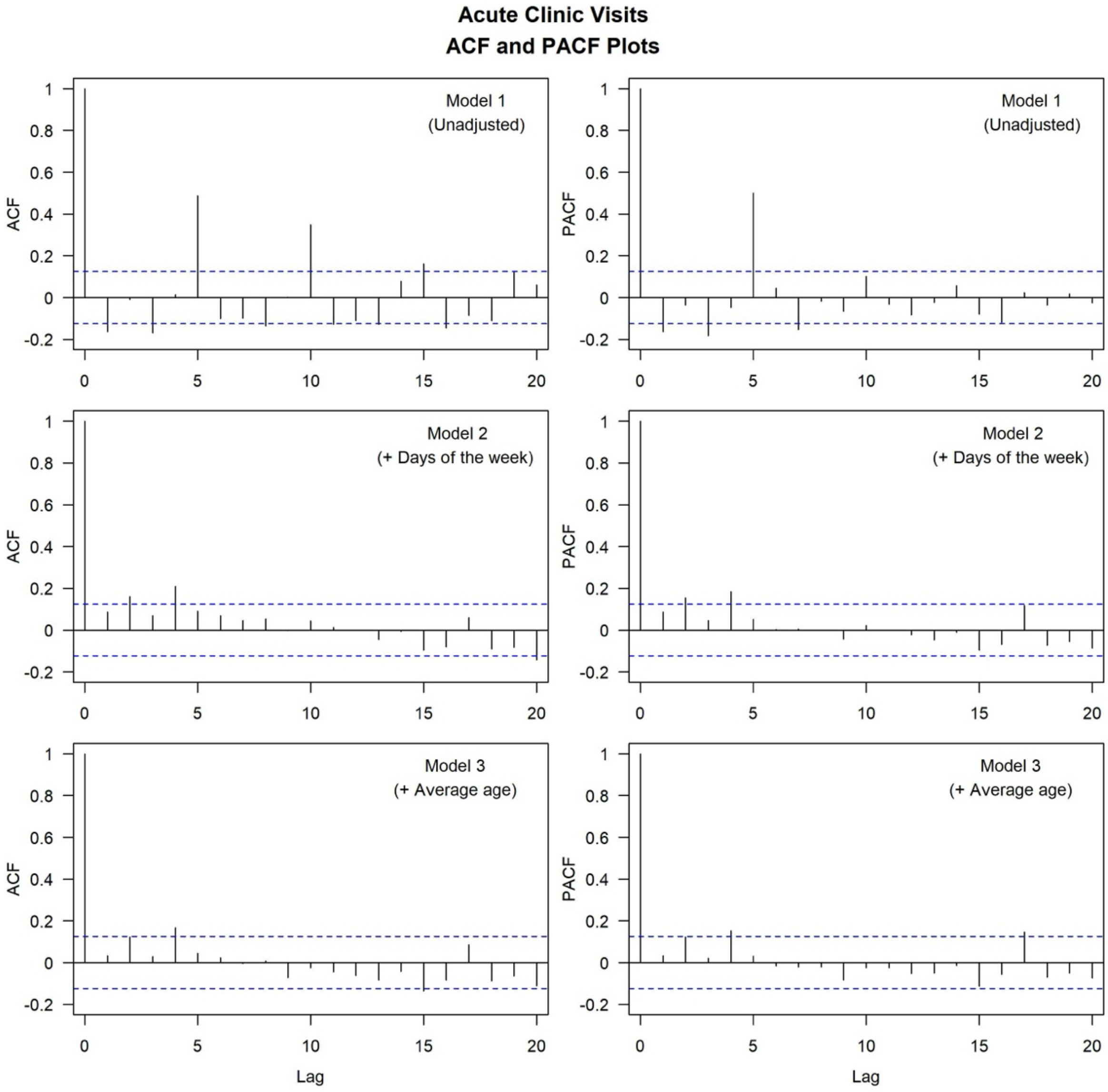

Supplementary Figure S3. Autocorrelation Function (ACF) and Partial Autocorrelation Function (PACF) plots for chronic clinic visits.

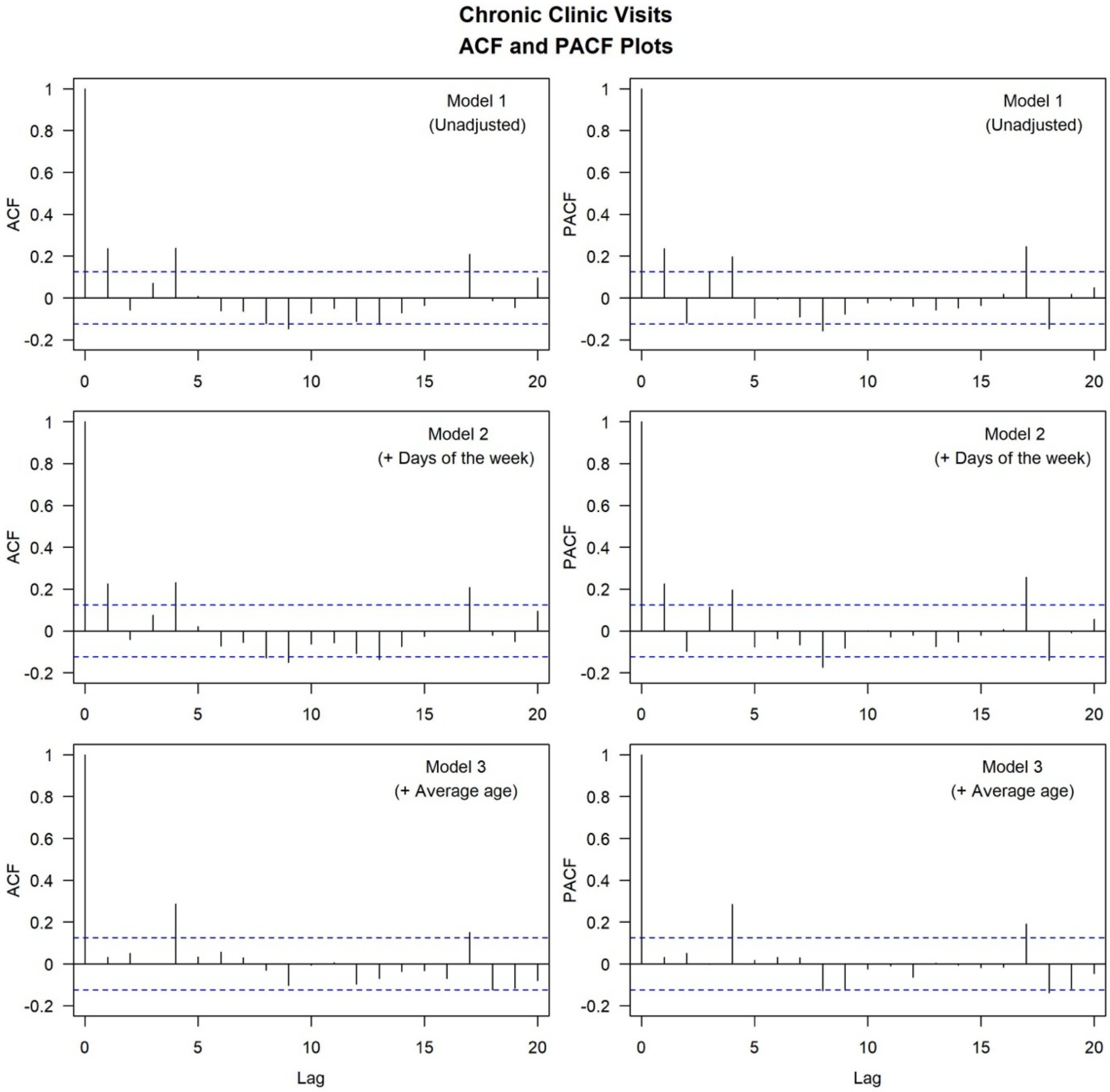

Supplement: Supplementary file 1 — Supplementary Information. [file 41598_2024_57142_MOESM1_ESM.pdf]
